# The long-term effects of the fenestration in patients with extracardiac Fontan circulation—a multicenter Korean cohort study based on national Fontan registry

**DOI:** 10.3389/fcvm.2024.1341882

**Published:** 2024-05-07

**Authors:** Hoon Ko, Jinyoung Song, Sang Ah Chi, Sang-Yun Lee, Soo-Jin Kim, Chang-Ha Lee, Chun Soo Park, Eun Seok Choi, Hyo Soon An, I. Seok Kang, Ja Kyoung Yoon, Jae-Suk Baek, Jae-Young Lee, Joowon Lee, June Huh, Kyung-Jin Ahn, Se Yong Jung, Seul Gi Cha, Yeo-Hyang Kim, Young-Seok Lee

**Affiliations:** ^1^ Department of Pediatrics, Pusan National University Yangsan Hospital, Pusan National University School of Medicine, Yangsan, Republic of Korea; ^2^ Department of Pediatrics, Samsung Medical Center, Sungkyunkwan University School of Medicine, Seoul, Republic of Korea; ^3^ Department of Health Sciences and Technology, Samsung Advanced Institute for Health Sciences & Technology, Sungkyunkwan University, Seoul, Republic of Korea; ^4^ Biomedical Statistics Center, Samsung Medical Center, Research Institute for Future Medicine, Seoul, Republic of Korea; ^5^ Department of Pediatrics, College of Medicine, Seoul National University Children’s Hospital, Seoul, Republic of Korea; ^6^ Department of Pediatrics, Sejong General Hospital, Bucheon, Republic of Korea; ^7^ Department of Thoracic and Cardiovascular Surgery, Sejong General Hospital, Bucheon, Republic of Korea; ^8^ Department of Thoracic and Cardiovascular Surgery, Asan Medical Center, University of Ulsan College of Medicine, Seoul, Republic of Korea; ^9^ Department of Pediatrics, Asan Medical Center, University of Ulsan College of Medicine, Seoul, Republic of Korea; ^10^ Department of Pediatrics, College of Medicine, Seoul St. Mary’s Hospital, The Catholic University of Korea, Seoul, Republic of Korea; ^11^ Department of Pediatrics, College of Medicine, Seoul National University Bundang Hospital, Sungnam, Republic of Korea; ^12^ Department of Pediatric Cardiology, Gachon University Gil Medical Center, Incheon, Republic of Korea; ^13^ Department of Pediatrics, Severance Cardiovascular Hospital, Yonsei University College of Medicine, Seoul, Republic of Korea; ^14^ Department of Pediatrics, Kyungbook National University School of Medicine, Daegu, Republic of Korea; ^15^ Department of Pediatrics, Dong-A University Hospital, Busan, Republic of Korea

**Keywords:** Fontan, fenestration, outcome, registries, propensity score

## Abstract

**Introduction:**

The long-term effects of fenestration in patients with Fontan circulation remain unclear. We aim to evaluate the fenestration impact on early and late outcomes in patients with extracardiac Fontan (ECF) using a propensity score matching analysis.

**Methods:**

We performed an extensive retrospective multicenter clinical data review of the Korean Fontan registry and included 1,233 patients with surgical ECF (779 fenestrated, 454 non-fenestrated). Demographics, baseline, and follow-up data were collected and comprehensively analyzed. Patients were divided into two groups according to the baseline presence or absence of surgical fenestration. Subsequently, patients were sub-divided according to the fenestration status at the last follow-up. Propensity-score matching was performed to account for collected data between the 2 groups using a multistep approach. The primary outcomes were survival and freedom from Fontan failure (FFF). We also looked at postoperative hemodynamics, cardiopulmonary exercise test results, oxygen saturations, and functional status.

**Results:**

After propensity-score matching (454 matched pairs), there was no difference in survival or FFF between the 2 groups. However, ECF patients with baseline fenestration had significantly lower oxygen saturation (*p *= 0.001) and lower functional status (*p *< 0.001). Patients with fenestration had significantly longer bypass times, higher postoperative central venous pressure, higher postoperative left atrial pressure, and less prolonged pleural effusion in the early postoperative period. The propensity score matching according to the fenestration status at the last follow-up (148 matched pairs) showed that patients with a persistent fenestration had significantly lower oxygen saturation levels (*p *< 0.001). However there were no intergroup differences in the functional status, survival and FFF.

**Conclusions:**

Our results showed no long-term benefits of the Fenestration in terms of survival and FFF. Patients with persistent fenestration showed oxygen desaturation but no difference in exercise intolerance was shown between the 2 groups.

## 1 Introduction

The original Fontan operation was described by Francis Fontan in 1971 for a patient with tricuspid atresia (TA) (1); since then, some modifications have been developed for a functional single ventricular heart. Extracardiac conduit Fontan (ECF) can be fenestrated and not fenestrated, and fenestrated Fontan operations are now the most commonly used modifications. Owing to its simplicity and superior surgical outcomes (2–5), ECF has been accepted as the primary choice in Korea. However, controversy remains regarding fenestration. Fenestrated Fontan, which was first proposed for high-risk patients in 1990 (6), has demonstrated some benefits in terms of pleural drainage, hospital length of stay, and short-term morbidity (7, 8). However, it allows some degree of arterial desaturation and carries the possibility of systemic thromboembolism such as stroke (8). Thompson et al. concluded that fenestration was not necessary in all patients undergoing ECF (9). However, exercise intolerance in patients after ECF is well known (10, 11). Long-term Fontan-associated liver disease (FALD), a serious consequence after Fontan operation, has recently recognized after the Fontan operation (12–14). Fontan fenestration is expected to reduce pressure in the Fontan circuit and increase cardiac output by increasing the preload (9). These might be reasons why some still insist on fenestration in ECF. Previous studies assessing the necessity and impact of the fenestration were limited by their small number of patients and lack of randomization. The long-term effects of fenestration in patients with Fontan circulation remain unclear. Therefore, we aim to evaluate the fenestration impact on early and late outcomes in patients with ECF using a propensity score matching analysis.

## 2 Materials and methods

**Figure 1:**
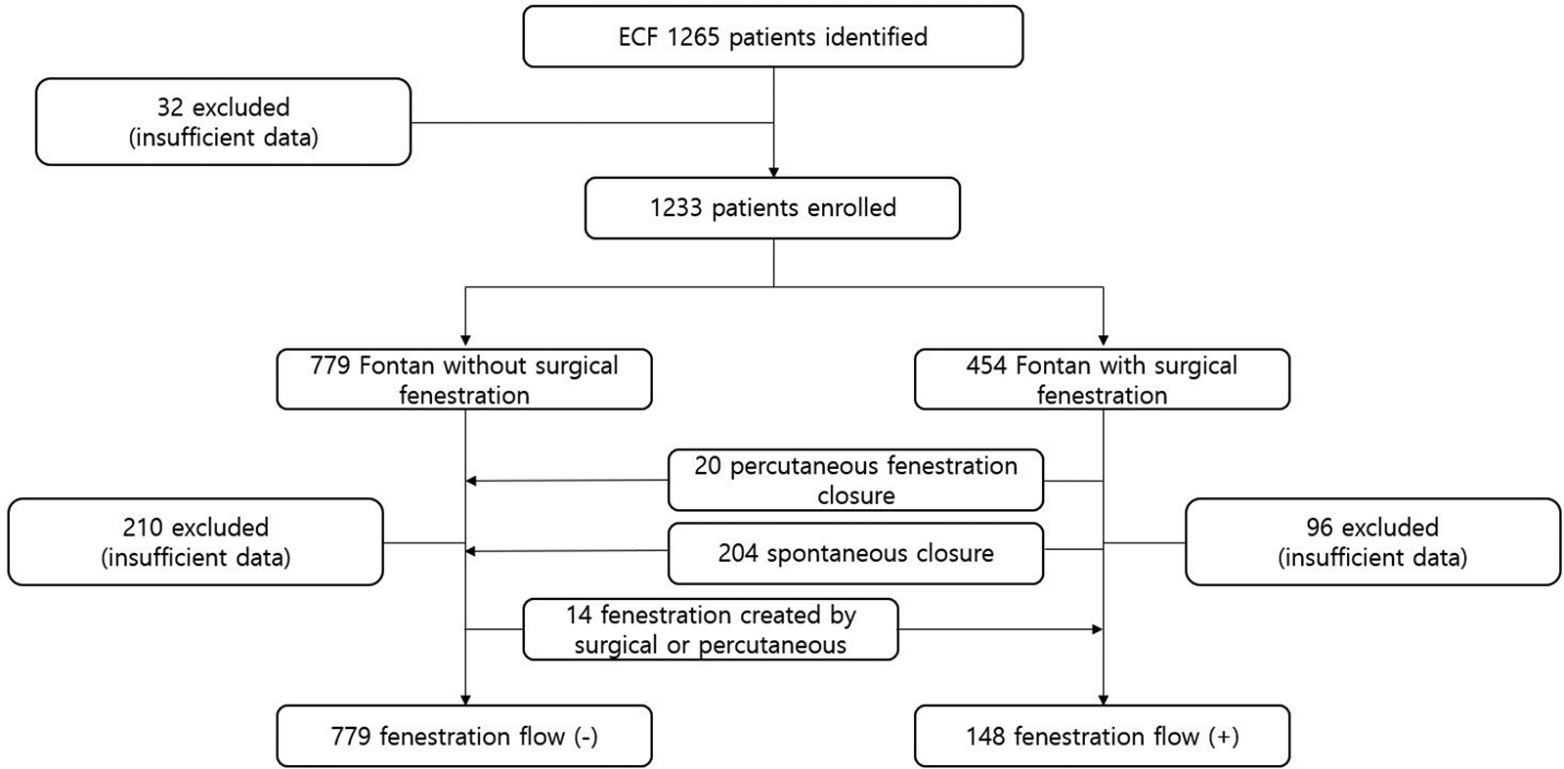
The study flow chart.

For the study, we performed an extensive clinical data review of the Korean Fontan registry and included 1,233 patients with surgical ECF (779 fenestrated, 454 non-fenestrated). Demographics, baseline, and follow-up data were collected and comprehensively analyzed. The Korean Fontan registry is the first national multicenter registry for patients with congenital heart disease in Korea. The registry includes the medical records of all patients who underwent ECF surgeries between June 1988 and December 2019 in South Korea. The decision to fenestrate the ECF was made either systematically according to the institutional approach or by the team in charge according to each individual case. Patients were divided into two groups according to the baseline presence or absence of surgical fenestration. Subsequently, patients were sub-divided according to the fenestration status at the last follow-up. The primary outcomes were survival and freedom from Fontan failure (FFF). The study flow chart is summarized in patients who underwent ECF with or without surgical fenestration (Figure 1). We also looked at postoperative hemodynamics, cardiopulmonary exercise test results, oxygen saturations, and functional status at the last follow-up.

### 2.1 Definitions

Transthoracic echocardiography (TTE) was used to evaluate the status of Fontan fenestration during follow-up. Early mortality was defined as death occurring within the first 30 postoperative days after Fontan surgery. Prolonged pleural drainage was defined as chest drainage for >14 days postoperative or readmission for pleural effusion. Fontan failure was defined as death, takedown, transplantation, or protein-losing enteropathy (PLE). Liver cirrhosis was defined as the advanced irreversible stage of progressive hepatic fibrosis characterized by distortion of hepatic architecture and formation of regenerative nodules (15). Clinically significant arrhythmia was defined as an arrhythmia requiring antiarrhythmic medication, cardioversion/defibrillation, or pacing devices. Heart failure was defined as presence of current or prior characteristic symptoms, such as dyspnea and fatigue, and evidence of ventricular dysfunction as a cause of these symptoms (16). Functional status was evaluated at every follow-up visit using the NYHA classification (17). Cardiopulmonary exercise test (CPET) was performed on a treadmill or on an ergometer cycle (18). Patients performed a symptom-limited maximal exercise test using an incremental protocol that allowed reaching exhaustion. Peak oxygen consumption (peak VO2) was defined as the maximal oxygen consumption.

### 2.2 Statistical analysis

All statistical analyses were performed using R statistical software (version 3.6.3; Foundation for Statistical Computing, Vienna, Austria). A two-sample *t*-test or Wilcoxon rank-sum test was used as appropriate to examine continuous variables. The *χ*^2^ test or Fisher's exact test was used to examine categorical variables. Categorical variables were reported as frequency and percentage and continuous variables were represented as median with interquartile range (IQR). Statistical analysis for continuous variables was conducted using the Mann–Whitney *U*-test. And for categorical variables, the *χ*^2^ test or Fisher's exact test was conducted. Statistical significance was set at a two-sided *p*-value < 0.05.

To reduce the effects of selection bias and confounding factors, we used propensity score matching to estimate the effects of baseline presence/absence of surgical fenestration and the fenestration status at the last follow-up using a multistep approach (19) (Supplementary Figure S1).

In first step, missing values were imputed using multiple imputation by chained equations to generate five imputed datasets (20). To predict each variable with missingness, the following variables were used in the imputation model: pre-Fontan pulmonary artery pressure, pre-Fontan transpulmonary pressure gradient (TPG), pre-Fontan pulmonary resistance (Rp). The distributions of these variables varied across the five imputed datasets.

Then, propensity score matching was applied within each imputed dataset using 1:1 nearest-neighbor matching without replacement in the second step (21). No caliper was applied. The propensity score was calculated using logistic regression with the following matching variables: gender, age at the time of the Fontan operation, TA, mitral atresia (MA), double-inlet left ventricle (DILV), unbalanced atrioventricular septal defect (AVSD), hypoplastic left heart syndrome (HLHS), prior shunt, prior banding, prior atrioventricular valve repair, prior total anomalous pulmonary venous return (TAPVR) repair, prior aortic arch repair, bilateral bidirectional cavopulmonary shunt (BCPS), pre-Fontan pulmonary artery pressure (PAP), pre-Fontan TPG, pre-Fontan Rp, and concomitant procedure during the Fontan operation. For each matched dataset, the absolute standard mean difference (ASMD) was computed to assess the balance of the variables used for matching and confirm whether the value of each variable was less than 0.2 with love plots. Group comparison was also compared using Wilcoxon signed-rank test for continuous variables and the McNemar test for categorical variables.

In third step, generalized linear models were used to determine the effect of having baseline fenestration or fenestration at the last follow-up in each matched dataset. Linear regression models, logistic regression models and cox regression models were used to calculate β estimates, odds ratios (ORs), and hazard ratios (HRs) with 95% confidence intervals (CIs) of having fenestration. Lastly, the results were pooled from five imputed datasets using Rubin's rules (22).

## 3 Results

**Figure 2:**
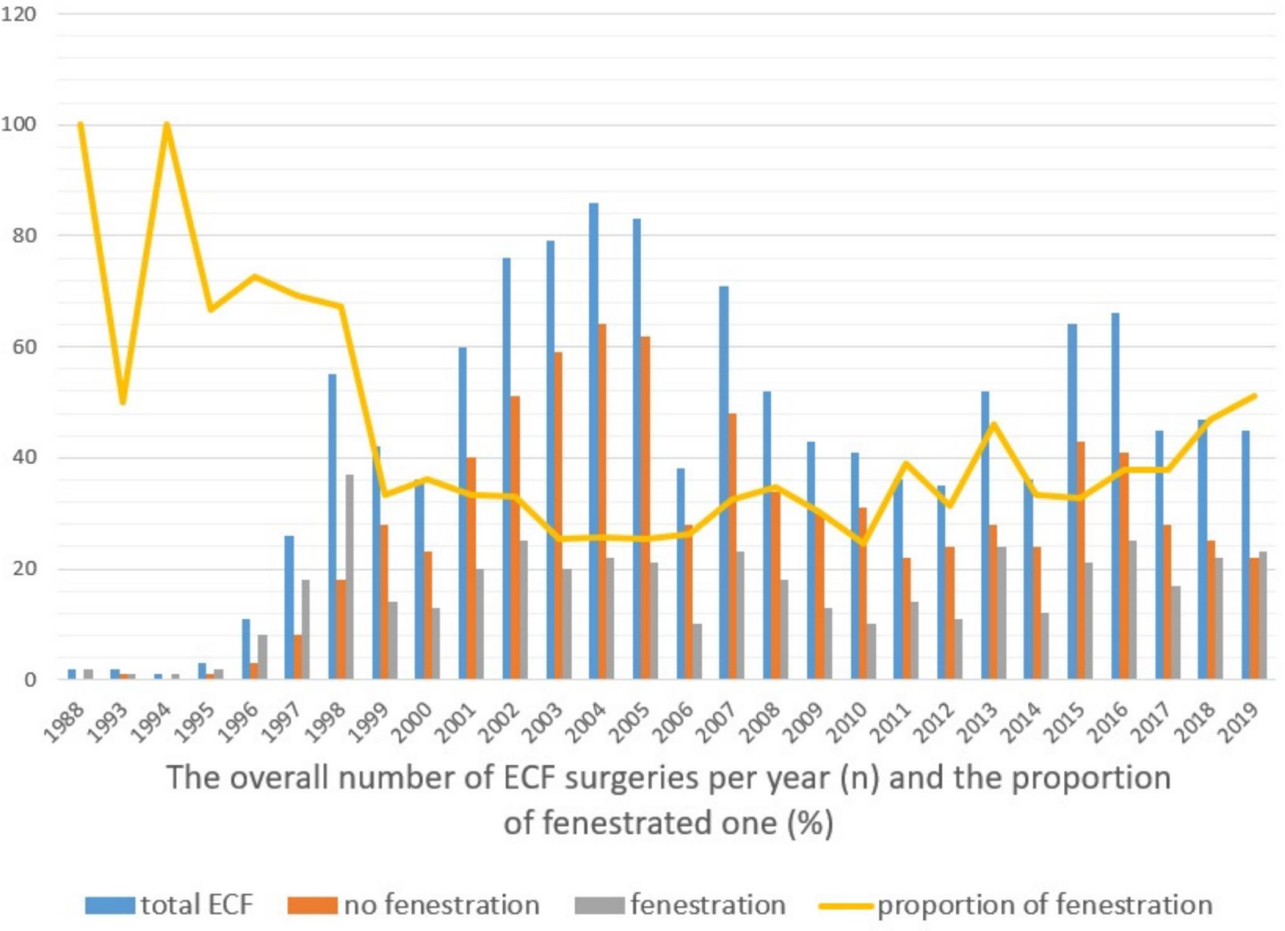
The overall number of ECF surgeries per year and the proportion of fenestrated one.

Among the enrolled cohort of Fontan survivors, the overall proportion of surgical fenestrations was 36.8% in 1988–2019 (Figure 2). After 2011, the proportion of Fontan procedures including surgical fenestration was approximately 40%. At the time of cross-sectional testing (median years after Fontan 12.8 years, IQR 5.4–18.1 years), the fenestration remained open in 41.3% (*n* = 148) of subjects.

### 3.1 Propensity score matching results

Among 1,233 patients with surgical ECF, there were 454 patients with baseline fenestration and 779 patients without baseline fenestration. The clinical, procedural, and follow-up characteristics in 1,233 patients were shown in Supplementary Tables S1–S3.

As there were 148 (12.0%), 339 (27.5%), and 313 (25.4%) missing values of the overall cohort for mean PAP, TPG, and Rp, respectively, we imputed these values by multiple imputation ad generated five imputed datasets. For each imputed dataset, 454 patients without baseline fenestration was matched with those with baseline fenestration (i.e., total of 908 patients in after-matched cohort). The 325 patients who were unmatched had significantly higher proportion of not having HLHS (*p *< 0.001), not having prior shunt (*p *< 0.001), and not having prior banding (*p *= 0.002) and not having concomitant procedure during Fontan operation (*p *< 0.001) and lower level of pre-Fontan PAP, pre-Fontan TPG, pre-Fontan Rp (all *p *< 0.001). The difference in these variables results into lower level of probability of getting baseline fenestration (i.e., propensity score), which would be a reason of being excluded from propensity score matching. After matching, all ASMDs for the matching variables were less than 0.2, which indicates well-balanced between two groups (Supplementary Figure S2).

### 3.2 Propensity score–matched 454 pairs according to baseline fenestration status

The baseline demographics and clinical characteristics of the matched cohort are shown in Table 1. Among a total of 908 patients, there were 538 males (59.3%) with median age at Fontan of 3.2 years. Median duration from Fontan to last follow-up was 10.7 years (IQR 4.4–15.7 years). No significant difference in all variables were found between patients with and without fenestration.

**Table 1:** Propensity score–matched 454 pairs according to baseline fenestration status.

Variable	Overall, *n* = 908	Fenestration, *n* = 454	No fenestration, *n* = 454	ASMD^a^	*p*-value
Demographics					
Male, *N* (%)	538 (59.3)	272 (59.9)	266 (58.6)	0.009	0.736
Age at Fontan (years), median (IQR)	3.2 (2.7, 4.1)	3.1 (2.6, 4.2)	3.3 (2.8, 4.1)	0.058	0.232
Duration from Fontan to last follow-up (years), median (IQR)	10.7 (4.4, 15.7)	10.8 (3.9, 16.7)	10.5 (4.6, 15.1)	0.001	0.584
Anatomy					
TA, *N* (%)	155 (17.1)	78 (17.2)	77 (17)	0.006	0.929
MA, *N* (%)	47 (5.2)	25 (5.5)	22 (4.8)	0.058	0.662
DILV, *N* (%)	67 (7.4)	34 (7.5)	33 (7.3)	0.008	0.900
Unbalanced AVSD, *N* (%)	75 (8.3)	37 (8.1)	38 (8.4)	0.040	0.901
HLHS, *N* (%)	68 (7.5)	36 (7.9)	32 (7)	0.049	0.593
Pre Fontan procedure					
Prior shunt, *N* (%)	384 (42.3)	193 (42.5)	191 (42.1)	0.009	0.890
Prior PAB, *N* (%)	208 (22.9)	104 (22.9)	104 (22.9)	0.052	1.000
Prior AV valve repair, *N* (%)	14 (1.5)	6 (1.3)	8 (1.9)	0.019	0.593
Prior TAPVR repair, *N* (%)	19 (2.1)	9 (2)	10 (2.2)	0.032	0.808
Prior aortic arch repair, *N* (%)	31 (3.4)	17 (3.7)	14 (3.1)	0.046	0.564
Pre Fontan hemodynamics					
Mean PA pressure (mmHg), median (IQR)	12 (9, 14)	12 (9, 15)	12 (9, 14)	0.086	0.581
TPG (mmHg), median (IQR)	5 (4, 6)	5 (4, 6)	5 (4, 6)	0.078	0.865
Rp (WU^a^m^2^), median (IQR)	1.7 (1.2, 2.2)	1.7 (1.2, 2.3)	1.6 (1.1–2.2)	0.126	0.073
Fontan operative characteristics					
Concomitant procedure, *N* (%)	383 (42.2)	195 (43)	188 (41.4)	0.040	0.592

As the representative dataset of matched cohort, the fifth imputed dataset was used to report the summary statistics of each variable with *P*-value to compare the distribution of each variable.

ASMD, absolute value of standardized mean difference; AV, atrioventricular; AVSD, atrioventricular septal defect; DILV, double inlet left ventricle; HLHS, hypoplastic left heart syndrome; IQR, interquantile range; MA, mitral atresia; PA, pulmonary artery; PAB, pulmonary artery banding; Rp, pulmonary resistance; TA, tricuspid atresia; TAPVR, total anomalous pulmonary vein return; TPG, transpulmonary gradient.

^a^ ASMD is calculated, which is maximum ASMD among five ASMDs calculated from five matched datasets.

Univariable and multivariable analyses for estimating the effect of baseline fenestration on operative, postoperative, follow-up outcomes were presented in Table 2. In univariable analyses, patients with fenestration had longer CPB time (β coefficient 25.592, 95% CI 16.962–34.222), higher post bypass CVP (*β* coefficient 0.733, 95% CI 0.221–1.244), higher post bypass LAP (*β* coefficient 3.119, 95% CI 0.68–5.558), less prolonged pleural effusion (OR 0.658, 95% CI 0.476–0.909) for operative and postoperative outcomes in the early postoperative period. For follow-up outcomes, higher NYHA class (OR 2.905, 95% CI 1.795–4.7 for 2 vs. 1 and OR 8.07, 95% CI 1.03–63.252 for 3 vs. 1) and lower oxygen saturation (*β* coefficient −2.379, 95% CI −3.451 to 1.307) were found to be significant in favor of ECF patients with fenestration. After adjusting all matching variables, having baseline fenestration was still significantly associated with these variables.

**Table 2:** The effect of baseline fenestration on various outcomes in the matched 454 pairs.

Outcome	Univariable analysis				Multivariable analysis^a^			
	Estimate	95% LCL	95% UCL	*p*-value	Estimate	95% LCL	95% UCL	*p*-Value
Fontan operative data								
CPB time^b^	25.592	16.962	34.222	<0.001	24.052	16.001	32.103	<0.001
ACC time^b^	4.072	−2.294	10.439	0.211	3.809	−2.478	10.095	0.237
Postoperative SBP (mmHg)^b^	3.989	−0.803	8.782	0.112	4.144	−0.557	8.846	0.093
Postoperative CVP (mmHg)^b^	0.733	0.221	1.244	0.005	0.716	0.198	1.233	**0**.**007**
Postoperative LAP (mmHg)^b^	3.119	0.68	5.558	0.015	2.907	0.294	5.521	**0**.**035**
Postoperative data								
Prolonged pleural effusion^c^	0.658	0.476	0.909	0.013	0.637	0.456	0.889	**0**.**010**
Early mortality^c^	1.745	0.64	4.76	0.278	2.287	0.668	7.828	0.189
Follow-up hemodynamic data								
CVP^b^	−0.502	−1.508	0.504	0.330	−0.470	−1.498	0.558	0.374
VEDP^b^	−0.238	−1.648	1.171	0.742	−0.449	−1.902	1.005	0.553
TPG^b^	0.292	−0.493	1.076	0.468	0.358	−0.489	1.206	0.413
Qs^b^	0.306	−0.098	0.71	0.141	0.165	−0.271	0.601	0.463
Rp^b^	−0.059	−0.344	0.226	0.685	−0.128	−0.454	0.199	0.450
Follow-up CPET								
Peak VO_2_ (ml/kg/m^2^)^b^	0.096	−1.825	2.018	0.922	0.316	−1.377	2.010	0.716
Predictive peak VO_2_^b^	0.886	−3.329	5.101	0.683	0.918	−2.986	4.821	0.647
RER^b^	−0.021	−0.061	0.02	0.318	−0.019	−0.059	0.022	0.368
Late outcome								
NYHA class								
2 vs. 1^c^	2.905	1.795	4.7	0.001	3.176	1.924	5.242	**<0.001**
3 vs. 1^c^	8.07	1.03	63.252	0.049	11.174	1.884	66.288	**0.011**
4 vs. 1^c^	3.E + 04	0.001	1.E + 12	0.346	2.E + 05	2.E-06	3.E + 16	0.389
Oxygen saturation (%)^b^	−2.379	−3.451	−1.307	0.001	−2.354	−3.426	−1.282	**0**.**001**
Fontan takedown^c^	1.05	0.191	5.782	0.955	0.841	0.106	6.655	0.870
Heart transplantation^c^	0.525	0.146	1.883	0.324	0.402	0.103	1.566	0.190
Mortality^c^	1.048	0.62	1.774	0.860	1.043	0.603	1.804	0.880
PLE^d^	0.584	0.293	1.164	1.000	0.541	0.266	1.098	1.000
Fontan failure^c^	0.837	0.543	1.291	0.423	0.800	0.503	1.273	0.349
Systemic thromboembolism^c^	1.351	0.611	2.989	0.459	1.285	0.568	2.908	0.547
Stroke^c^	1,486.774	4.E-06	6.E + 11	0.538	1.E + 11	9.E-33	2.E + 54	0.750
Liver cirrhosis^c^	1.176	0.701	1.974	0.541	1.179	0.663	2.096	0.578
Arrhythmia^c^	1.107	0.661	1.853	0.701	1.114	0.641	1.938	0.702
Heart failure^c^	0.982	0.414	2.329	0.967	0.926	0.357	2.4	0.874

ACC, aortic cross clamp time; AVSD, atrioventricular septal defect; BCPS, bidirectional cavopulmonary shunt; CPB, cardiopulmonary bypass; CPET, cardiopulmonary exercise test; CVP, central venous pressure; DILV, double inlet left ventricle; HLHS, hypoplastic left heart syndrome; LAP, left atrial pressure; MA, mitral atresia; NYHA, New York Heart association; PAP, pulmonary artery pressure; PLE, protein losing enteropathy; Qs, systemic blood flow; RER, respiratory exchange rate; Rp, pulmonary resistance; SBP, systolic blood pressure; TA, tricuspid atresia; TPG, transpulmonary gradient; VEDP, ventricular end diastolic pressure, VO_2_, oxygen consumption.

Significant *p*-values are given in bold.

^a^ Adjusted for all matching variables (i.e., gender, age at Fontan, TA, MA, DILV, unbalanced AVSD, HLHS, prior shunt, prior banding, prior atrioventricular valve repair, prior total anomalous pulmonary vein return repair, prior aortic arch repair, bilateral BCPS, pre-Fontan PAP, pre-Fontan TPG, pre-Fontan Rp and concomitant procedure during Fontan operation).

^b^ *β* coefficient and 95% CIs for having baseline fenestration are calculated via linear regression model.

^c^ ORs and 95% CIs for having baseline fenestration are calculated via logistic regression model.

^d^ HRs and 95% CIs for having baseline fenestration are calculated via cox proportional hazards regression model.

### 3.3 Propensity score–matched 148 pairs according to fenestration status at last follow-up

Out of 1,233 patients with 779 without surgical fenestration and 454 surgical fenestration, 210 and 96 patients were excluded for insufficiency of data. Then, 238 patients were also excluded from the analysis of fenestration status at last follow-up because of specific reasons below.

#### 3.3.1 Catheter closed fenestration

Among the patient underwent Fontan procedure with surgical fenestration (*n* = 454), excluding 96 patients with insufficient data, 5.6% (*n* = 20) underwent transcatheter fenestration closure with covered stents or closure devices. The following criteria were assessed to determine suitability for fenestration closure: an unobstructed Fontan pathway with no significant decompressing venovenous collaterals, baseline Fontan pressure <15 mmHg, baseline cardiac index >2l/min/m^2^, and decrease in cardiac index <20% with test occlusion of the fenestration (23).

If the parameters for closure suitability were met, the fenestration was closed. All children received prophylactic antibiotics and anticoagulant according to institutional protocol. After device closure, patients resumed routine anticoagulation or antiplatelet agent according to the primary cardiologist's preference.

#### 3.3.2 Spontaneous fenestration closure

Among the patient underwent Fontan procedure with surgical fenestration (*n* = 454), excluding 96 patients with insufficient data and 20 patient with transcatheter fenestration closure, 60.4% (*n* = 204) experienced apparent spontaneous closure at the last follow-up. As the current medications in patients who are being followed, most patients are taking anticoagulation and or antithrombotic regimen (overall cohort 94.7%, anticoagulation 38.3%, and antithrombotic regimen 59.6%).

#### 3.3.2 Fenestration re-creation

Fourteen patients without initial surgical fenestration underwent surgical or percutaneous fenestration after the Fontan operation. Indications for recreated fenestration included severe chronic effusions or PLE.

#### 3.3.3 Propensity score–matched 148 pairs according to fenestration status at last follow-up

With remained 927 patients with 148 patients (16%) for open fenestration, there were 102 (11.0%), 239 (25.8%), and 214 (23.1%) missing values of the overall cohort for mean PAP, TPG, and Rp, respectively. After multiple imputation for these missing values, 148 patients without fenestration at last follow-up were matched with those with fenestration with last follow-up for each imputed dataset (i.e., total of 296 patients in after-matched cohort). The 631 patients who were unmatched had significantly higher proportion of not having HLHS (*p* < 0.001), not having prior shunt (*p* < 0.001), not having prior banding (*p* = 0.002), not having prior TAPVR repair (*p* = 0.038) and not having concomitant procedure during Fontan operation (*p* < 0.001), and lower level of pre-Fontan TPG (*p* = 0.030) and pre-Fontan Rp (*p* < 0.001). The difference in these variables results into lower level of probability of getting fenestration at last follow-up (i.e., propensity score), which would be a reason of being excluded from propensity score matching. After matching, all ASMDs for the matching variables were less than 0.2, which indicates well-balanced between two groups (Supplementary Figure S3). The baseline demographics and clinical characteristics of the before-matched and after-matched cohorts are shown in Supplementary Table S4.

In before-matched group, there were significantly higher proportion of HLHS, prior shunt, and concomitant procedure and higher level of Rp in the open fenestration group. These results suggest that patients in the present fenestration flow group had higher disease severity at the time of the Fontan operation than those in the absent fenestration flow group. After propensity score matching, there were no significant difference in all variables including HLHS, prior shunt, Rp, and concomitant procedure. The follow-up characteristics for after-matched cohort were presented in Table 3. Only oxygen saturation level was significantly different between open fenestration and no fenestration groups.

**Table 3:** Propensity score–matched 148 pairs according to fenestration status at last follow-up.

Variable^a^	Overall, *n* = 296	Open fenestration, *n* = 148	No fenestration, *n* = 148	*p*-value
Follow up hemodynamic data				
CVP (mmHg), median (IQR)	13 (10.5, 15)	14 (11, 16)	12.5 (10, 15)	0.131
VEDP (mmHg), median (IQR)	10 (7, 12)	9 (6, 12)	10 (7.8, 12)	0.365
TPG (mmHg), median (IQR)	4 (3, 6)	4 (4, 6)	4 (2, 5.5)	0.491
Qs (ml/min/m^2^), median (IQR)	3.5 (2.5, 4.3)	3.7 (2.6, 4.8)	3.3 (2.4, 3.9)	0.250
Rp (WU^a^m^2^), median (IQR)	1.1 (0.9, 1.8)	1.1 (0.9, 1.7)	1.2 (0.8, 1.9)	0.437
Follow up CPET				
Peak VO_2_ (ml/kg/m^2^), median (IQR)	27 (22.5, 31.3)	25.1 (21.1, 30.1)	27.9 (23, 31.4)	0.626
Predictive peak VO_2_ (%), median (IQR)	56 (46.8, 66)	51 (41.2, 67)	57 (50.3, 64.8)	0.777
RER, median (IQR)	1.1 (1, 1.2)	1 (1, 1.1)	1.1 (1, 1.2)	0.925
Late outcome				
NYHA class, *N (%)*				0.414
1	229 (77.4)	111 (75)	118 (79.7)	
2	50 (16.9)	28 (18.9)	22 (14.9)	
3	5 (1.7)	4 (2.7)	1 (0.7)	
4	2 (0.7)	1 (0.7)	1 (0.7)	
Oxygen saturation (%), median (IQR)	92 (88, 95)	89 (86, 92.5)	94 (92, 95)	**<0**.**001**
Fontan takedown, *N (%)*	0 (0)	0 (0)	0 (0)	NA^b^
Heart transplantation, *N (%)*	3 (1)	1 (0.7)	2 (1.4)	0.564
Mortality, *N (%)*	9 (3)	5 (3.4)	4 (2.7)	0.739
PLE, *N (%)*	16 (5.4)	9 (6.1)	7 (4.7)	0.593
Fontan failure, *N (%)*	25 (8.4)	13 (8.8)	12 (8.1)	0.827
Systemic thromboembolism, *N (%)*	10 (3.4)	4 (2.7)	6 (4.1)	0.527
Stroke, *N (%)*	1 (0.3)	0 (0)	1 (0.7)	0.317
Liver cirrhosis, *N (%)*	34 (11.5)	12 (8.1)	22 (14.9)	0.083
Arrhythmia, *N (%)*	23 (7.8)	9 (6.1)	14 (9.5)	0.371
Heart failure, *N (%)*	6 (2)	4 (2.7)	2 (1.4)	0.414

As the representative dataset of matched cohort, the fifth imputed dataset was used to report the summary statistics of each variable with *P*-value to compare the distribution of each variable.

CPET, cardiopulmonary exercise test; CVP, central venous pressure; IQR, interquantile range; NYHA, New York Heart association; PLE, protein losing enteropathy; Qs, systemic blood flow; RER, respiratory exchange rate; Rp, pulmonary resistance; TPG, transpulmonary gradient; VEDP, ventricular end diastolic pressure, VO2, oxygen consumption.

Significant *p*-values are given in bold.

^a^ There exist missing values in data: 197 (66.6%), 203 (68.6%), 219 (74%), 232 (78.4%), 224 (75.7%), 207 (69.9%), 208 (70.3%), 207 (69.9%), 10 (3.4%), 60 (20.3%), 18 (6.1%), 20 (6.8%), 18 (6.1%), 18 (6.1%), 19 (6.4%), 21 (7.1%), 18 (6.1%), and 19 (6.4%) of the overall cohort for CVP, VEDP, TPG, Qs, Rp, peak VO_2_, predictive peak VO_2_, RER, NYHA class, Oxygen saturation, Fontan takedown, Heart transplantation, PLE, Systemic thromboembolism, Stroke, Liver cirrhosis, Arrhythmia, and Heart failure, respectively.

^b^ NA indicates that *P*-value cannot be calculated because of zero Fontan takedown in both groups.

Univariable and multivariable analyses for estimating the effect of fenestration at last follow-up on follow-up outcomes were presented in Table 4. In univariable analyses, patients with fenestration at last follow-up had lower oxygen saturation level (*β* coefficient −4.073, 95% CI −5.311 to 2.836). After adjusting all matching variables, having fenestration at last follow-up was still significantly associated with lower oxygen saturation level (*β* coefficient −4.247, 95% CI −5.552 to 2.942). Other variables showed no significant association with fenestration at last follow-up.

**Table 4:** The effect of fenestration at last follow-up on various outcomes in the matched 148 pairs.

Outcome	Univariable analysis				Multivariable analysis^a^			
	Estimate	95% LCL	95% UCL	*p*-value	Estimate	95% LCL	95% UCL	*p*-value
Follow up hemodynamic data								
CVP(mmHg)^b^	0.907	−0.634	2.449	0.254	1.296	−0.164	2.756	0.089
VEDP(mmHg)^b^	−0.21	−2.001	1.581	0.820	0.746	−0.902	2.395	0.388
TPG(mmHg)^b^	0.846	−0.341	2.033	0.180	0.995	−0.33	2.32	0.180
Qs(ml/min/m^2^)^b^	0.707	−0.007	1.422	0.062	0.494	−0.183	1.17	0.182
Qp/Qs^b^	0.077	−0.167	0.321	0.538	0.157	−0.12	0.435	0.286
Rp (WU^a^m^2^)^b^	−0.186	−0.633	0.261	0.423	−0.047	−0.501	0.407	0.842
Follow up CPET								
Peak VO2(ml/kg/m2)^b^	−0.64	−4.122	2.841	0.720	0.075	−3.227	3.376	0.965
Predictive peak VO2 (%)^b^	−4.219	−11.035	2.598	0.231	−1.432	−9.288	6.424	0.726
RER^b^	−0.054	−0.124	0.016	0.145	−0.039	−0.129	0.05	0.415
Late outcome								
NYHA class								
2 vs. 1^c^	1.314	0.49	3.524	0.600	2.579	0.699	9.516	0.190
3 vs. 1^c^	3.432	0.314	37.483	0.316	3.E + 06	6.E-09	2.E + 21	0.421
4 vs. 1^c^	328.519	7.E-06	2.E + 10	0.578	6.E-05	2.E-16	2.E + 07	1.000
Oxygen saturation (%)^b^	−4.073	−5.311	−2.836	<0.001	−4.247	−5.552	−2.942	**<0**.**001**
Fontan takedown^c^	0.03	1.E-09	7.E + 05	0.704	0.018	7.E-11	5.E + 06	0.704
Heart transplantation^c^	0.617	0.038	9.947	0.735	0.005	5.E-11	4.E + 05	0.595
Mortality^c^	3.E + 03	4.E-06	2.E + 12	0.638	331.233	1.E-07	1.E + 12	0.629
PLE^c^	1.56	0.454	5.366	0.485	2.332	0.463	11.738	0.310
Fontan failure^c^	1.527	0.531	4.389	0.438	1.401	0.403	4.866	0.600
Systemic thromboembolism^c^	1.005	0.163	6.187	0.995	0.858	0.097	7.573	0.892
Stroke^c^	0.001	8.E-13	9.E + 05	0.542	0.003	4.E-11	3.E + 05	0.574
Liver cirrhosis^c^	0.676	0.289	1.58	0.370	1.371	0.295	6.376	0.692
Arrhythmia^c^	0.716	0.276	1.858	0.494	0.867	0.227	3.310	0.837
Heart failure^c^	1.847	0.255	13.401	0.548	2.E-05	4.E-21	1.E + 11	0.588

AVSD, atrioventricular septal defect; BCPS, bidirectional cavopulmonary shunt; CPET, cardiopulmonary exercise test; CVP, central venous pressure; DILV, double inlet left ventricle; HLHS, hypoplastic left heart syndrome; MA, mitral atresia; NYHA, New York Heart association; PAP, pulmonary artery pressure; PLE, protein losing enteropathy; Qs, systemic blood flow; RER, respiratory exchange rate; Rp, pulmonary resistance; TA, tricuspid atresia; TAPVR, total anomalous pulmonary vein return; TPG, transpulmonary gradient; VEDP, ventricular end diastolic pressure; VO2, oxygen consumption.

Significant *p*-values are given in bold.

^a^ Adjusted for all matching variables (i.e., gender, age at Fontan, TA, MA, DILV, unbalanced AVSD, HLHS, prior shunt, prior banding, prior atrioventricular valve repair, prior TAPVR repair, prior aortic arch repair, bilateral BCPS, pre-Fontan PAP, pre-Fontan TPG, pre-Fontan Rp and concomitant procedure during Fontan operation).

^b^ *β* coefficient and 95% CIs for having fenestration at last follow-up are calculated via linear regression model.

^c^ ORs and 95% CIs for having fenestration at last follow-up are calculated via logistic regression model.

## 4 Discussion

The Korea Fontan registry is the first multicenter national registry of patients who underwent the Fontan operation in Korea. In our study, fenestration performed during ECF failed to provide any long-term benefits, including survival and Fontan failure, but led to systemic desaturation and a lower functional status. However, the benefits of early postoperative pleural drainage have been demonstrated. We observed that a considerable number of fenestrations closed spontaneously. Patients with persistent fenestration flow had no long-term benefits in terms of survival, FALD, exercise intolerance, and Fontan failure but had lower oxygen saturation than those without persistent fenestration flow.

Recently, a similar study of the Australia and New Zealand Fontan registry concluded that fenestration in the Fontan circulation had no early or long-term benefits but resulted in a higher incidence of thromboembolic events (24). In contrast to our study, the study of the ANZ Fontan registry included patients with lateral tunnel and ECF modifications, and it was unclear whether the fenestration represented persistent patent fenestration.

The early postoperative effect of fenestration reportedly reduces the length of hospital stay and duration of pleural drainage (8, 25–28). However, several studies demonstrated excellent outcomes without fenestration and improved oxygen saturation after fenestration (29–31). Therefore, fenestration should be limited to patients with high-risk Fontan circulation and enthusiastic transcatheter closure (9, 32). Still, there have been controversies on routine transcatheter closure of fenestration. Technically, transcatheter closure of fenestration in EC or lateral tunnel Fontan is not tricky. Not only various kinds of occlusive device but balloon expandable covered stent or self expanding graft stent have been used with good results (33–36). It has been reported improved systemic saturation and exercise capacity but no adverse events from several studies (29, 31, 37).

A few studies suggested that there may be some benefits to persistent fenestration. Saiki et al. demonstrated the chronic cardioprotective effect of persistent fenestration on long-term Fontan circulation (38). Greenleaf et al. reported a significant increase in Fontan pressure after fenestration closure (31), while Oka et al. reported that the absence of fenestration flow was a predictor of FALD (39).

A meta-analysis performed by Bouhout showed results very similar to those of our study, proving a shorter duration of pleural drainage but no long-term benefits (26). Unlike our study, another meta-analysis demonstrated no difference in oxygen saturation in patients with patent fenestration (40). Both analyses showed no long-term benefits or higher risk of stroke with fenestration (26, 40). Our patients with persistent fenestration had a lower oxygen saturation but no difference in exercise capacity and no history of stroke. Although the veno-venous collaterals (VVC) may affect systemic arterial desaturation, this study did not include an investigation into the prevalence and/or flow of the VVC.

There were changes in the tendency to perform fenestration during ECF. The lowest proportion of fenestrated ECF in Korea was 24.7% in 2004; this value gradually increased to 45.8% in 2018. The proportions of fenestrated ECF vs. non-fenestrated ECF differed among the participating institutes. There was only one institution performing routine Fontan perforations. The criteria for Fontan fenestration were not consistent as they differed according to the policies of each heart center. One of the main reasons for a consistent fenestrated ECF might be the concern of long-term Fontan failure, including FALD, although most studies to date failed to prove the long-term benefits of fenestration. Our data showed a 60.4% spontaneous closure rate of the initial fenestration. The spontaneous closure rate in our patients might be much higher than that reported by Gorla et al. (22%) (41) and hemodynamic data were not proven as significant factors for spontaneous closure in our analysis. However, fenestration type, size, and anticoagulation agents used are the most important factors. Owing to a lack of data, it was not possible to analyze these factors in our study.

The long-term incidence of systemic thromboembolic events in our analysis was lower than those in the ANZ Fontan registry (24). The definitions of systemic thromboembolic events differed between the two studies, and the lack of systemic screening for silent stroke in our study resulted in a lower stroke rate.

These retrospective studies including our study have their crucial limitations, so that a well-designed prospective study in homogenous group of patients should be conducted afterward. For a long-term effect, close observations with comprehensive evaluations for a long enough time are needed.

This study has limitations owing to its retrospective and multicenter design. The indication for Fontan fenestration, transcatheter fenestration closure and subsequent fenestration creation were not exactly consistent as they differed according to the policies of each heart center. The impact of a learning curve associated with surgical skills and postoperative care by individual surgeon and cardiologist may have affected the results of this study. This study is not an alternative to randomized control studies but is an important add-on to the literature.

## 5 Conclusion

Our results showed no long-term benefits of the Fenestration in terms of survival and FFF. Patients with persistent fenestration showed oxygen desaturation but no difference in exercise intolerance was shown between the 2 groups. ECF patients with baseline fenestration showed significantly reduced pleural drainage during the early postoperative period. Regarding the long-term effects of fenestrated ECF, our study showed similar findings to ECF patients with persistent fenestration but poorer functional class than those with baseline fenestration.

## Data Availability

The original contributions presented in the study are included in the article/**Supplementary Material**, further inquiries can be directed to the corresponding author.
