# Supplementary material for: The long-term effects of the fenestration in patients with extracardiac Fontan circulation—a multicenter Korean cohort study based on national Fontan registry
Source: Front Cardiovasc Med. 2024 May 7;11:1341882. doi: 10.3389/fcvm.2024.1341882 (PMC11106450; doi:10.3389/fcvm.2024.1341882)
Supplement: Supplementary file 1 [file Datasheet1.pdf]

Supplementary Table 1. Clinical characteristics in before-matched cohort by baseline fenestration status

Variable*	Overall, n=1233	Fenestration, n=454	No Fenestration, n=779	*p*-Value
**Demographics**				
Male, *N (%)*	726 (58.9)	272 (59.9)	454 (58.3)	0.616
Age at Fontan (yrs), median (IQR)	3.2 (2.7, 4)	3.1 (2.6, 4.2)	3.2 (2.8, 3.9)	0.423
Duration from Fontan to last follow-up (yrs), median (IQR)	10.4 (4.3, 15.6)	10.8 (3.9, 16.7)	10.2 (4.5, 15.2)	0.224
**Anatomy**				
TA, *N (%)*	203 (16.5)	78 (17.2)	125 (16)	0.661
MA, *N (%)*	74 (6)	25 (5.5)	49 (6.3)	0.664
DILV, *N (%)*	103 (8.4)	34 (7.5)	69 (8.9)	0.465
Unbalanced AVSD, *N (%)*	111 (9)	37 (8.1)	74 (9.5)	0.487
HLHS, *N (%)*	70 (5.7)	36 (7.9)	34 (4.4)	**0.013**
**Pre Fontan procedure**				
Prior shunt, *N (%)*	461 (37.4)	193 (42.5)	268 (34.4)	**0.006**
Prior PAB, *N (%)*	301 (24.4)	104 (22.9)	197 (25.3)	0.384
Prior AV valve repair, *N (%)*	17 (1.4)	6 (1.3)	11 (1.4)	1.000
Prior TAPVR repair, *N (%)*	27 (2.2)	9 (2.0)	18 (2.3)	0.859
Prior aortic arch repair, *N (%)*	51 (4.1)	17 (3.7)	34 (4.4)	0.705
**Pre Fontan hemodynamics**				
mean PA pressure (mmHg), median (IQR)	11 (9, 13)	12 (9, 15)	11 (8, 13)	**<0.001**
TPG (mmHg), median (IQR)	4 (3, 6)	5 (4, 6)	4 (3, 6)	**0.001**
Rp (WU*m^2), median (IQR)	1.5 (1, 2.1)	1.8 (1.2, 2.3)	1.4 (1, 1.9)	**<0.001**
**Fontan operative characteristics**				
Concomitant procedure, *N (%)*	438 (35.5)	195 (43)	243 (31.2)	**<0.001**

AV, atrioventricular; AVSD, atrioventricular septal defect; DILV, double inlet left ventricle; HLHS, hypoplastic left heart syndrome; IQR, interquantile range; MA, mitral atresia; PA, pulmonary artery; PAB, pulmonary artery banding; Rp, pulmonary resistance; TA, tricuspid atresia; TAPVR, total anomalous pulmonary vein return; TPG, transpulmonary gradient.

*There exist missing values in data: 148 (12.0%), 339 (27.5%), and 313 (25.4%) of the overall cohort for mean PA pressure, TPG, and Rp, respectively.

Supplementary Table 2. Procedural characteristics in before-matched cohort by baseline fenestration status

Variable*	Overall, n=1233	Fenestration, n=454	No Fenestration, n=779	*p*-Value
**Fontan operative data**				
CPB time (min), median (IQR)	115 (84, 154)	138 (103.5, 180)	105 (78, 141)	**<0.001**
ACC time (min), median (IQR)	35 (18, 58)	40 (19, 62)	31 (17, 54)	**0.036**
Post bypass SBP (mmHg), median (IQR)	82 (71, 94)	85 (73, 96)	8 (70, 92)	**<0.001**
Post bypass CVP (mmHg), median (IQR)	15 (13, 17)	16 (14, 18)	15 (13, 17)	**<0.001**
Post bypass LAP (mmHg), median (IQR)	9 (6, 11)	11 (9, 13)	7 (6, 10)	**<0.001**
**Post-operative data**				
Prolonged pleural effusion, *N (%)*	364 (29.5)	109 (24.0)	255 (32.7)	**0.002**
Early mortality, *N (%)*	17 (1.4)	10 (2.2)	7 (0.9)	0.101

ACC, aortic cross clamp time; CPB, cardiopulmonary bypass; CVP, central venous pressure; IQR, interquantile range; LAP, left atrial pressure; SBP, systolic blood pressure.

*There exist missing values in data: 101 (8.2%), 669 (54.3%), 110 (8.9%), 171 (13.9%), and 1112 (90.2%) of the overall cohort for CPB time, ACC time, Post bypass SVP, Post bypass CVP, and Post bypass LAP, respectively.

Supplementary Table 3. Follow-up characteristics in before-matched cohort by baseline fenestration status

Variable*	Overall, n=1233	Fenestration, n=454	No Fenestration, n=779	*p*-Value
**Follow up hemodynamic data**				
CVP (mmHg), median (IQR)	13 (11, 15)	13 (10, 15)	13 (11, 15.5)	0.522
VEDP (mmHg), median (IQR)	10 (7, 12)	10 (7, 12)	9 (7, 11)	0.609
TPG (mmHg), median (IQR)	4 (3, 5)	4 (3, 6)	4 (3, 5)	0.229
Qs(ml/min/m^2), median (IQR)	3.2 (2.4, 4)	3.3 (2.4, 4)	3.2 (2.5, 3.9)	0.518
Rp (WU*m^2), median (IQR)	1.4 (0.9, 1.9)	1.3 (0.9, 1.8)	1.4 (1, 1.9)	0.575
**Follow up CPET**				
peak VO_2 (ml/kg/m^2), median (IQR)	26.8 (21.9, 30.7)	26.4 (21.3, 30.6)	27.2 (22.1, 30.7)	0.579
predictive peak VO_2 (%), median (IQR)	58 (47, 68)	58 (46, 69)	57 (49, 67)	0.969
RER, median (IQR)	1.1 (1, 1.2)	1.1 (1, 1.2)	1.1 (1, 1.1)	0.829
**Late outcome**				
NYHA class, *N (%)*				**<0.001**
1	885 (71.8)	285 (62.8)	600 (77)	
2	138 (11.2)	87 (19.2)	51 (6.5)	
3	13 (1.1)	9 (2.0)	4 (0.5)	
4	5 (0.4)	3 (0.7)	2 (0.3)	
Oxygen saturation, median (IQR)	93 (91, 95)	92 (88, 95)	94 (92, 96)	**<0.001**
Fontan takedown, *N (%)*	6 (0.5)	3 (0.7)	3 (0.4)	0.815
Heart transplantation, *N (%)*	14 (1.1)	4 (0.9)	10 (1.3)	0.703
Mortality, *N (%)*	82 (6.7)	34 (7.5)	48 (6.2)	0.433
PLE, *N (%)*	57 (4.6)	17 (3.7)	40 (5.1)	0.337
Fontan failure, *N (%)*	133 (10.8)	49 (10.8)	84 (10.8)	1.000
Systemic thromboembolism, *N (%)*	32 (2.6)	16 (3.5)	16 (2.1)	0.163
Stroke, *N (%)*	3 (0.2)	2 (0.4)	1 (0.1)	0.668
Liver cirrhosis, *N (%)*	105 (8.5)	46 (10.1)	59 (7.6)	0.200
Arrhythmia, *N (%)*	82 (6.7)	36 (7.9)	46 (5.9)	0.191
Heart failure, *N (%)*	26 (2.1)	11 (2.4)	15 (1.9)	0.692

CVP, central venous pressure; CPET, cardiopulmonary exercise test; IQR, interquantile range; PLE, protein losing enteropathy; Qs, systemic blood flow; Rp, pulmonary resistance; TPG, transpulmonary gradient; VEDP, ventricular end diastolic pressure; VO_2, oxygen consumption.

*There exist missing values in data: 960 (77.9%), 965 (78.3%), 1023 (83%), 1055 (85.6%), 1034 (83.9%), 890 (72.2%), 898 (72.8%), and 895 (72.6%), 192 (15.6%), 458 (37.1%), 194 (15.7%), 196 (15.9%), 172 (13.9%), 174 (14.1%), 236 (19.1%), 269 (21.8%), 269 (21.8%), and 178 (14.4%) of the overall cohort for CVP, VEDP, TPG, Qs, Rp, peak VO2, predictive peak VO2, RER, NYHA class, Oxygen saturation, Fontan takedown, Heart transplantation, PLE, Systemic thromboembolism, Stroke, Liver cirrhosis, Arrhythmia, and Heart failure, respectively.

Supplementary Table 4. Clinical characteristics in before-matched and after-matched cohorts by fenestration status at last follow-up

Variable	Before-matched cohort, n= 927*			After-matched cohort, n= 296			
	Open Fenestration, n=148	No Fenestration, n=779	*p*-Value	Open Fenestration, n= 148	No Fenestration, n= 148	ASMD**	*p*-Value
**Demographics**							
Male, *N (%)*	90 (60.8)	468 (60.1)	0.9397	90 (60.8)	93 (62.8)	0.055	0.714
Age at Fontan (yrs), median (IQR)	3.1 (2.7, 3.8)	3.1 (2.7, 3.8)	0.6439	3.1 (2.7, 3.8)	3.1 (2.8, 4.1)	0.084	0.857
Duration from Fontan to last follow-up (yrs), median (IQR)	12.4 (5, 17.3)	11.7 (5.5, 15.8)	0.1115	7.3 (3, 14.1)	11.1 (4.7, 15.5)	-	**<0.001**
**Anatomy**							
TA, *N (%)*	23 (15.5)	140 (18)	0.5522	23 (15.5)	27 (18.2)	0.019	0.547
MA, *N (%)*	11 (7.4)	51 (6.5)	0.8291	11 (7.4)	14 (9.5)	0.077	0.549
DILV, *N (%)*	12 (8.1)	60 (7.7)	0.9987	12 (8.1)	8 (5.4)	0.099	0.346
Unbalanced AVSD, *N (%)*	13 (8.8)	62 (8)	0.8627	13 (8.8)	11 (7.4)	0.119	0.637
HLHS, *N (%)*	16 (10.8)	31 (4)	0.0011	16 (10.8)	17 (11.5)	0	0.782
**Pre Fontan procedure**							
Prior shunt, *N (%)*	71 (48)	285 (36.6)	0.0118	71 (48)	66 (44.6)	0.081	0.475
Prior PAB, *N (%)*	40 (27)	191 (24.5)	0.5871	40 (27)	39 (26.4)	0.030	0886
Prior AV valve repair, *N (%)*	3 (2)	11 (1.4)	0.8456	3 (2)	6 (4.1)	0.048	0.317
Prior TAPVR repair, *N (%)*	5 (3.4)	17 (2.2)	0.5607	5 (3.4)	6 (4.1)	0.037	0.739
Prior aortic arch repair, *N (%)*	4 (2.7)	33 (4.2)	0.5192	4 (2.7)	2 (1.4)	0.083	0.414
**Pre Fontan hemodynamics**							
mean PA pressure (mmHg), median (IQR)	12 (8, 14.8)	11 (9, 13)	0.1223	11.5 (8, 14.3)	11 (8, 13.3)	0.091	0.595
TPG (mmHg), median (IQR)	4.8 (4, 6)	4 (3, 6)	0.3456	5 (4, 6)	5 (3, 6)	0.062	0.579
Rp (WU*m^2), median (IQR)	1.6 (1.2, 2.4)	1.4 (1, 2)	0.0103	1.5 (1.2, 2.3)	1.5 (1.1, 2.1)	0.036	0.196
**Fontan operative characteristics**							
Concomitant procedure, *N (%)*	64 (43.2)	264 (33.9)	0.0368	64 (43.2)	70 (47.3)	0.136	0.460

As the representative dataset of after-matched cohort, the fifth imputed dataset was used to report the summary statistics of each variable with P-value to compare the distribution of each variable.

AV, atrioventricular; AVSD, atrioventricular septal defect; DILV, double inlet left ventricle; HLHS, hypoplastic left heart syndrome; IQR, interquantile range; MA, mitral atresia; PA, pulmonary artery; PAB, pulmonary artery banding; Rp, pulmonary resistance; TA, tricuspid atresia; TAPVR, total anomalous pulmonary vein return; TPG, transpulmonary gradient.

*There exist missing values in data: 102 (11.0%), 239 (25.8%), and 214 (23.1%) of the before-matched cohort for mean PA pressure, TPG, and Rp, respectively.
**ASMD is calculated, which is maximum ASMD among five ASMDs calculated from five matched datasets. ASMD=absolute value of standardized mean differenc

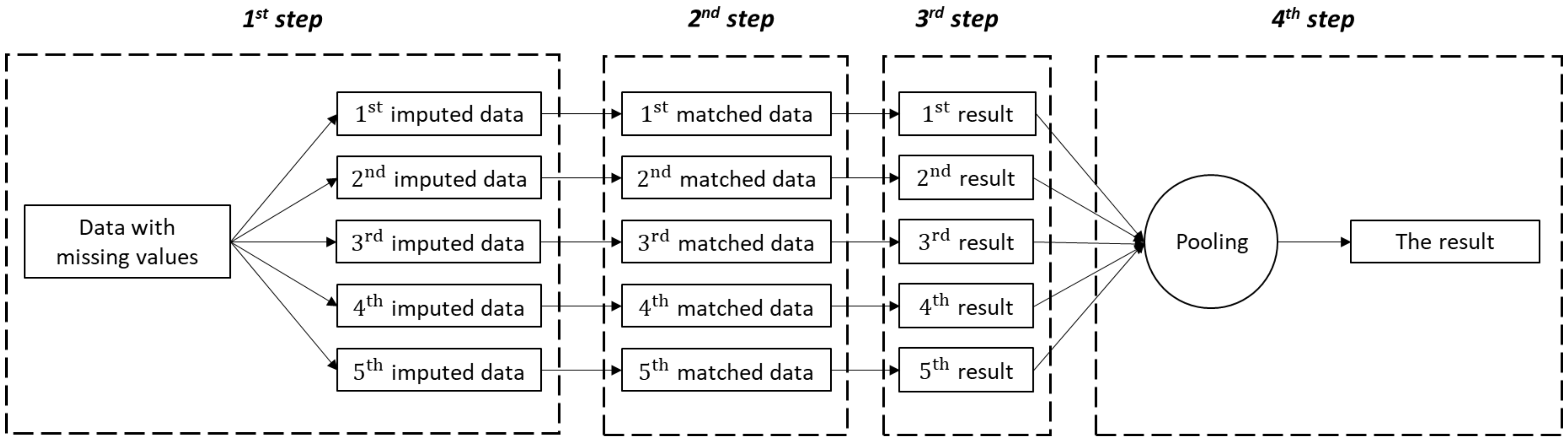

Supplementary Figure 1. Graphical presentation of the propensity score matching method after multiple imputation

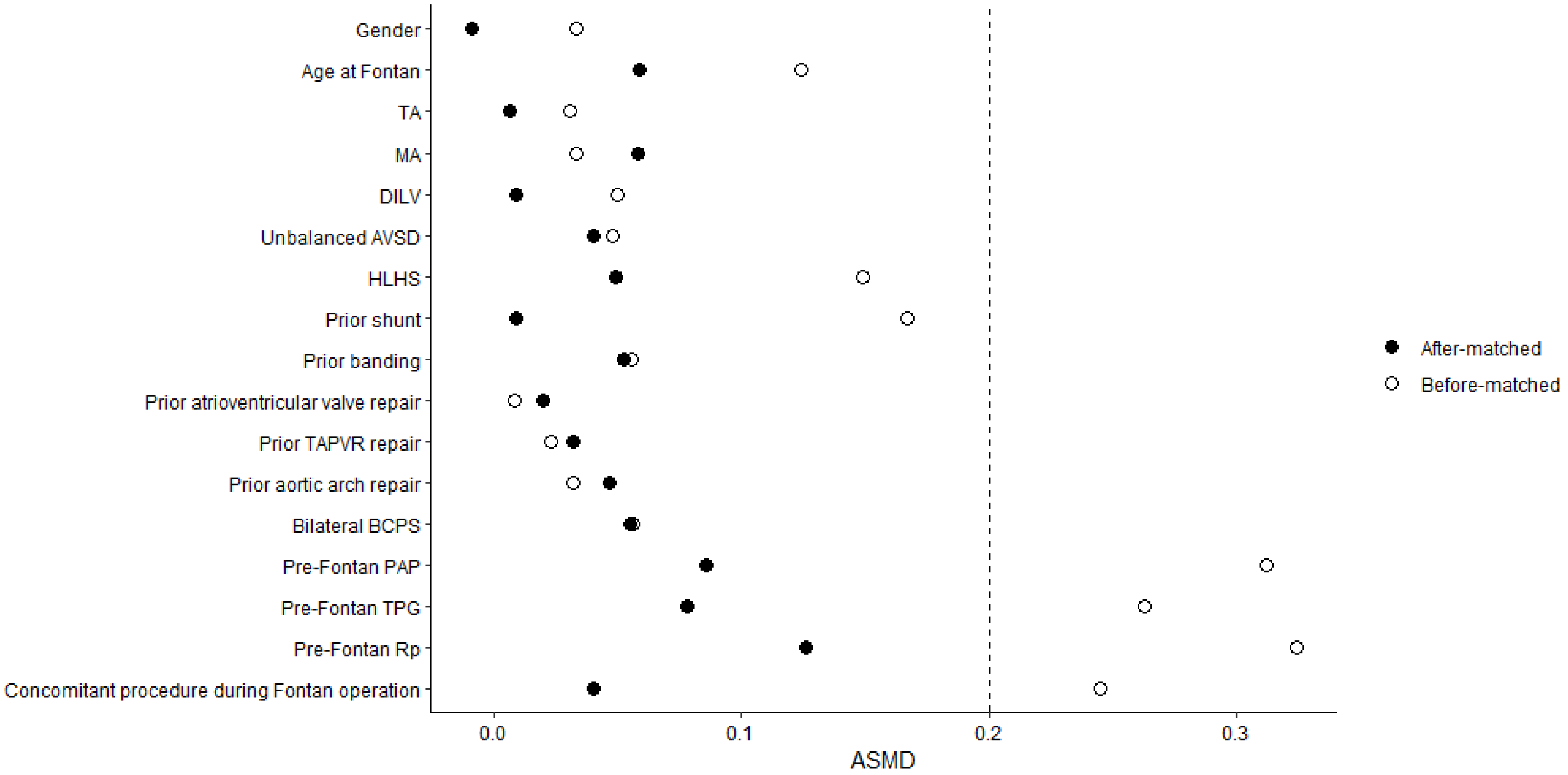

Supplementary Figure 2. Love plot for matching variables between before-matched and after-matched cohort after propensity score matching on baseline fenestration. As the representative dataset of after-matched cohort, the fifth imputed dataset was used to report the ASMD of each variable to assess the balance between two groups.

TA, tricuspid atresia; MA, mitral atresia; DILV, double inlet left ventricle; AVSD, atrioventricular septal defect; HLHS, hypoplastic left heart syndrome; TAPVR, total anomalous pulmonary vein return; BCPS, bidirectional cavopulmonary shunt; PAP, pulmonary artery pressure; TPG, transpulmonary pressure gradient; Rp, pulmonary resistance.

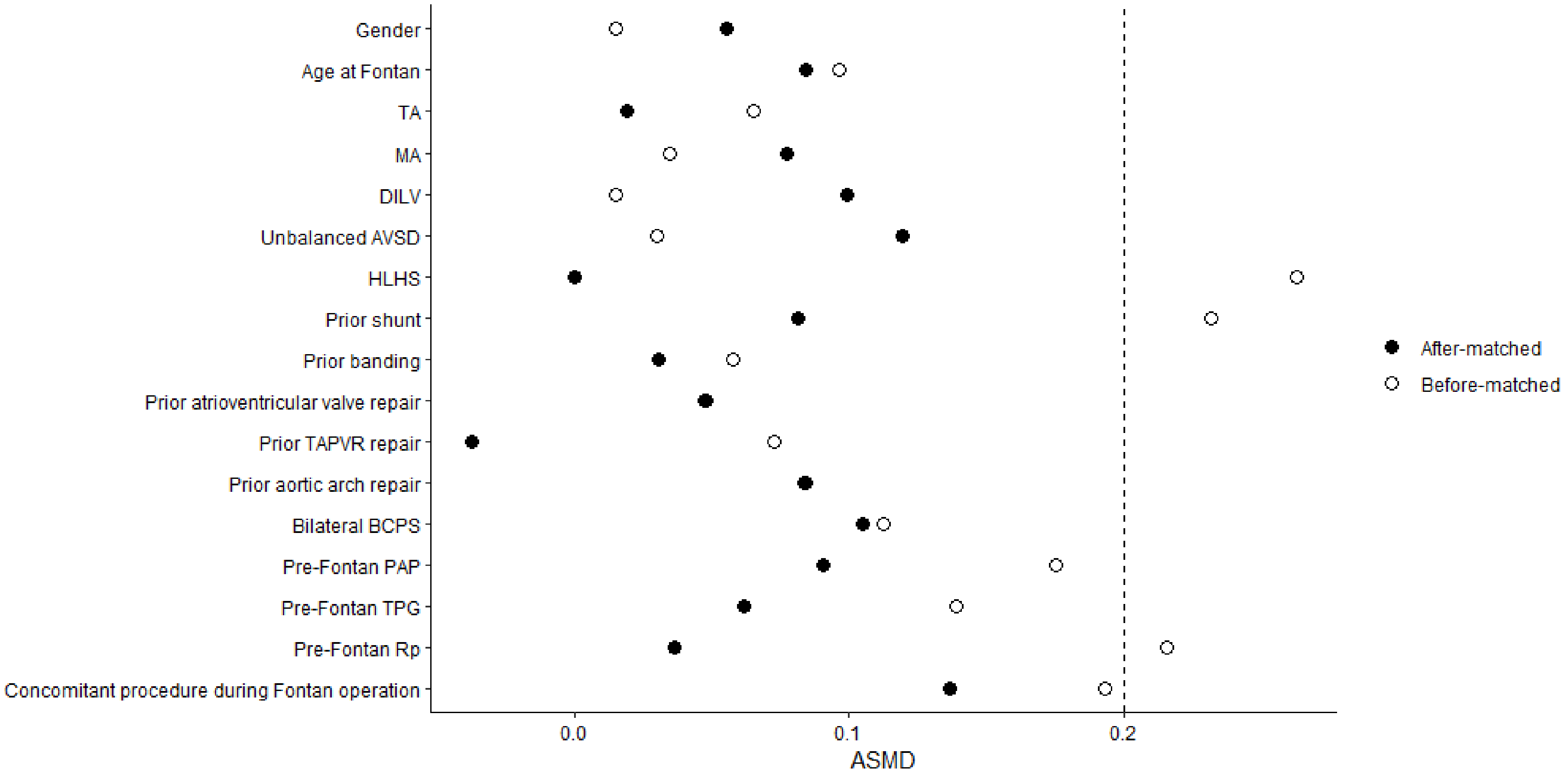

Supplementary Figure 3. Love plot for matching variables between before-matched and after-matched cohort after propensity score matching on fenestration at last follow-up. As the representative dataset of after-matched cohort, the fifth imputed dataset was used to report the ASMD of each variable to assess the balance between two groups.

TA, tricuspid atresia; MA, mitral atresia; DILV, double inlet left ventricle; AVSD, atrioventricular septal defect; HLHS, hypoplastic left heart syndrome; TAPVR, total anomalous pulmonary vein return; BCPS, bidirectional cavopulmonary shunt; PAP, pulmonary artery pressure; TPG, transpulmonary pressure gradient; Rp, pulmonary resistance.
